# John Dee and the sciences: early modern networks of knowledge

**DOI:** 10.1016/j.shpsa.2011.12.001

**Published:** 2012-09

**Authors:** Jennifer M. Rampling

**Affiliations:** University of Cambridge, Department of History and Philosophy of Science, Free School Lane, Cambridge CB2 3RH, UK

## 1 Dee and demarcation

The significance of John Dee (1527–1609) for historians of science rests both on the range of his interests and activities, and the problems this range has caused for his biographers. A mathematician learned in British history, cartography, astrology and navigation, throughout his life Dee also became increasingly engaged with alchemy, kabbalah, divination, and communion with spirits. All of these interests, and more, were represented in his library, one of the largest and most comprehensive in Europe, particularly for scientific content. The range of his pursuits was famously taxonomised in his account of ‘the Sciences, and Artes Mathematicall’ in the *Mathematicall praeface* to Henry Billingsley’s English translation of Euclid’s *Elements* (1570). Here, Dee’s ‘Groundplat’ of the sciences laid out the applications of geometry and arithmetic not only in the fields of mathematics and natural philosophy, but also in ‘thinges Supernaturall, æternall, & Diuine.’^1^

Historiographically, Dee’s Protean interests have proved no easier to classify than his spectra of the mathematical arts. Dee’s esoteric pursuits were well known to his contemporary detractors, and continue to occupy the popular imagination today, yet the academy has struggled to reconcile his natural philosophical interests with those apparently antithetical to modern conceptions of science. In the absence of any single, outstanding contribution to support his inclusion in canonical histories of the Scientific Revolution, for much of the twentieth century Dee remained a marginal figure in broader histories of science.^2^ His re-engineering as a hermetic magus in the 1960s and 70s offered a new narrative, in which Dee became the case study *par excellence* for the influence of Neoplatonic currents on sixteenth- and seventeenth-century developments in astronomy and natural philosophy. The synthetic studies of I. R. F. Calder (1952), Peter French (1972) and Frances Yates (1969, 1972, 1979), in emphasising Dee’s occult philosophical interests, thereby provided a sharp contrast with the internalist histories of E. G. R. Taylor (1930, 1954), Francis Johnson (1937) and John Heilbron (1978), who, in focusing on Dee’s contributions to mathematics, geography, astronomy and navigation, distinguished these enquiries from his occult leanings.^3^

Contradictory anatomies of Dee as either ‘scientist’ or ‘magus’ provided the impetus for Nicholas Clulee’s seminal intellectual biography, *John Dee’s natural philosophy: between science and religion* (1988). Clulee challenged earlier interpretations of Dee ‘as an embodiment of some pre-existent intellectual tradition’ (1988, p. 3), instead tracing the evolution of Dee’s thought throughout his life. This endeavour was augmented by Julian Roberts and Andrew Watson’s reconstruction of *John Dee’s library catalogue* (1990), a bibliographical feat which revealed not only the scope and scale of Dee’s library, but also of his manuscript writings and annotations in printed books. Dee’s lesser studied compositions and marginalia also provided the focus for William Sherman’s influential monograph, *John Dee: the politics of reading and writing* (1995). By reevaluating Dee’s activities within the wider context of Elizabethan patronage, commerce and learned expertise, Sherman refocused attention on his active role as courtier and political and commercial ‘intelligencer.’ Dee’s occult interests were also revisited in the work of Deborah Harkness, who approached his apocalyptic, angelic and alchemical thinking as a sustained development of his natural philosophy, rather than an intellectual aberration, while situating the whole range of his activities within the domestic economy of his house at Mortlake (Harkness, 1999, 1997).

As early modern political circumstances changed, so did contemporary distinctions between legitimate and suspect activity. Dee’s struggles to find a stable position upon the lurching political and confessional map of Tudor England and Counter-Reformation Europe are foregrounded in Glyn Parry’s recent biography, *The arch-conjuror of England: John Dee* (2011). Parry follows Dee’s efforts—not always successful—to downplay or distract attention from his association with ‘conjuring’, in a series of attempts to safeguard his reputation and improve his prospects for preferment. In doing so, he reassesses Dee in the context of increased interest in alchemy, astrology and angel magic in European courts.

Such scholarly treatments, supplemented by interdisciplinary colloquia held in London, Szeged and Aarhus,^4^ and a London symposium on Dee’s alchemy,^5^ have contributed not only to a richer and more historically sensitive understanding of Dee’s achievements, but also to a new appreciation of the complexity and interconnectedness of the Elizabethan sciences. The contours of this historiographical journey are surveyed in Stephen Clucas’s detailed introduction to the essays in *John Dee: interdisciplinary studies in English Renaissance thought* (2006b). In perusing the range of Dee’s interests, from navigation to kabbalah, the collection underlines both the essentially interdisciplinary nature of early modern knowledge, and the extent to which Dee’s importance to intellectual historians lies in the very breadth of interest and expertise that has so often exasperated his later commentators. ‘Increasingly’, Clucas notes, ‘Dee’s “problematic” career has been explained, rather than explained away’ (2006b, p. 9).

The contributions in this special issue are based on papers presented at an international conference held in 2009 to commemorate the quatercentenary of Dee’s death, fittingly hosted by the first of his *almae matres*, St John’s College.^6^ In presenting the fruits of these modern-day exchanges, this volume re-evaluates John Dee as a natural philosopher deeply implicated in the courtly, intellectual and religious culture of his age. Seeking patronage and the freedom to pursue his own intellectual agenda, Dee dispensed advice to royal and aristocratic patrons, confederates and friends: tutoring in mathematics and navigation, advising Elizabeth I’s Privy Council on calendrical reform, providing politically apposite horoscopes, and offering angelically-mediated counsel to the Holy Roman Emperor. In mapping these complex networks, the studies in this volume reveal the social, political, and religious contexts within which the Elizabethan sciences were practised, the routes through which they were disseminated, and the uses to which they were applied. Dee’s own networks provide our groundplan for charting the definition, discussion and practice of the sciences in early modern Europe.

## 2 Dee in context

John Dee was born into a matrix of familiar, courtly and mercantile ties that were instrumental in shaping his subsequent fortunes. His father, Rowland Dee, bridged the worlds of court and commerce, serving as a steward (‘antesignanus dapiferorum’) to Henry VIII, and later as ‘King’s packer’.^7^ Although Rowland’s career ended in both financial and political disgrace, by sending John to university at Cambridge he equipped his son with the means of acquiring the education and social connections necessary to pursue his own career at court. Rowland’s membership of the Mercers’ Company of London also paved the way for John’s admittance by patrimony to the Company in 1555, a connection which facilitated his access to merchants involved in trading ventures, such as the Loks, who would later employ his geographical and nautical knowledge (Roberts, 2004). From his mother, on the other hand, John Dee received his house and land at Mortlake, conveniently close to Richmond Palace, which would subsequently serve as a site for his library, museum and alchemical workshops; as a venue for receiving the Queen and other visitors; and as security for loans and mortgages.^8^

Through his antiquarian studies, Dee pushed this genealogical network backwards into Britain’s legendary past. By claiming descent from King Arthur (after whom he named his son) and the Welsh royal family, Dee linked his own prehistory with that of his major patron, Elizabeth I.^9^ Through a similar process, Dee constructed Elizabeth’s own descent from the Welsh prince Madog, and hence her title to Madog’s discoveries in the New World. Such connections might provide either a validating underlay for outright claims to territory, or implied grounds for recognition and preferment. In the meantime, Dee’s family connections offered more physical and immediate access to sources of patronage, such as the relationship of his second wife, Jane Fromonds, or Fromoundes (1555–1605), to Elizabeth’s friend Katherine Carey Howard.^10^

The essays by Nicholas Clulee and Stephen Pumfrey chart Dee’s various attempts to integrate his vision within existing patronage structures. While seeking direct support from the Queen and others, Dee also acquired clients and pupils of his own. His household and library at Mortlake provided the hub of a scholarly network which, as Nicholas Clulee argues, he dreamed of expanding into an institution analogous to a modern ‘research institute’, capable of recruiting international scholarship in the national interest. However, his plans did not always chime with the views of cash-strapped princes and suspicious prelates. Dee never succeeded in obtaining rewards commensurate with his own estimation of his intellectual attainments.

Dee laid out these attainments in his apologetic *Compendious rehearsal*, written in 1592 for royal commissioners appointed by Elizabeth I to investigate his pecuniary distress – part of which was subsequently published to counter popular accusations of conjuring. Here, Dee recounts his education and early promise, from his arrival, aged 15, at St. John’s College, where, adopting a contemporary scholarly trope, he described himself as having studied for eighteen hours a day (Dee, 1851, p. 5). In 1546, Dee was elected a fellow and under-reader in Greek at the newly founded Trinity College, and the following year spent his Cambridge vacation at the University of Louvain, where he met ‘with some learned men, and chiefely mathematicians, as Gemma Frisius, Gerardus Mercator, Gaspar à Mirica, Antonio Gogava, &c.’^11^ Still in his early twenties, he was in Paris by the summer of 1550, where his lectures on Euclid, he claimed, caused a sensation, resulting in offers of employment from the French King and the University of Paris (Dee, 1726, p. 526). In the *Rehearsal*, Dee stressed his connections with some of the most prominent names in European scholarship: Oronce Finé (1494–1555), Girolamo Cardano (1501–1576), Gemma Frisius (1508–1555), Federico Commandino (1509–1575), Gerardus Mercator (1512–1594), Petrus Ramus (1515–1572), Abraham Ortelius (1527–1598), and many more.

Dee’s relationships with contemporary mathematicians and cosmographers are examined in essays by Bruno Almeida and Stephen Johnston. Almeida returns to Dee’s formative years in England, Louvain and Paris, examining the role of the Portuguese cosmographer Pedro Nunes (1502–1578) in shaping Dee’s mathematical thought. In the absence of surviving correspondence between the two men, Almeida uses Dee’s marginal annotations and nautical writings to trace Nunes’ influence not only on the technical aspects of his work on loxodromic navigation, but on his larger goal of establishing navigation as a mathematical discipline. Johnston takes the opposite tack, using a recently discovered letter to shed light on the contents of Dee’s *Tyrocinium mathematicum* (1559), a now-lost mathematical work probably written for his pupil, Thomas Digges (1546–1595), and dedicated to Nunes. Arguing that this was the source for several of Dee’s annotations to Book X of Billingsley’s English *Euclid* (1570), Johnston draws attention to an aspect of Dee’s contribution that has long been overshadowed by the better known *Mathematicall praeface*.

As these studies remind us, the fields of Renaissance geometry and applied mathematics were far from static: punctuated by technical disputes, pedagogical innovations, and arguments over the status, scope and utility of mathematics. Dee probably accessed Nunes’ *Tratado da sphera* not from the original Portuguese, but via a Latin translation included in Diogo de Sá’s polemical attack on Nunes’ work. Dee’s comments and ‘advices’ to Book X of Euclid also respond to a recent controversy: Ramus’s criticism of Euclidean geometry, particularly Book X.

That Dee’s own views on geometry were more Neoplatonic than Ramist in character appears from the *Mathematicall praeface*, in which he cites the noble-born Italian philosopher Giovanni Pico della Mirandola (1463–1494). As Jean-Marc Mandosio shows, Dee followed Pico in his conception of ‘formal’ numbers: Platonic forms distinct from the quotidian world of ‘thinges *Numerable*.’ These exemplary numbers offered solutions to questions of universal and pressing importance, such as ‘When the world will end’ (on 1 January 2000, by Pico’s calculation). While Pico disdained the use of mathematics for quotidian numbering, dismissing Euclidean mathematics on these grounds, Dee drew no such distinction. In the *Praeface*, the eternal and diurnal dimensions of numbering provide a continuum within a ‘more general art Mathematical’: a programme perhaps better suited to the utilitarian ethos of English royal patronage than Pico’s more exclusive numerical hierarchy.^12^

Within this general art, Dee’s enigmatic references to the ‘Science Alnirangiat’ and another ‘OPTICAL science’ point to his underlying interest in natural magic and divination.^13^ Dee also devoted considerable space to a defence and definition of astrology, a field in which his expertise was at least as highly regarded as his knowledge of other areas of applied mathematics. Indeed, the paradoxical nature of Dee’s role at court is vividly illustrated by the use made of this knowledge by Elizabeth and Burghley. When it was politically expedient to do so, both drew upon Dee’s astrological expertise, although Dee was not always able to reap the benefits of such short-term favour, as Parry’s contribution to this volume reveals. When the crises passed, suspicion of Dee’s ‘conjuring’ history resurfaced, opponents renewed their criticisms, and his credit plummeted. There is a parallel here with his religious affiliations. Ordained as a priest during the reign of Mary Tudor, probably for reasons of expediency (Parry, 2011, pp. 28–29), Dee subsequently found it difficult to shake off the stigma of Catholicism under her successor. Opportunities for advancement were further circumscribed by the fact that Dee struggled to compete for posts with candidates whose orthodoxy had not been compromised. As Pumfrey’s essay shows, several clients who succeeded where Dee failed were able to boast more respectable Protestant credentials, having earlier suffered imprisonment, or fled Marian England into exile in the Low Countries. Connections, once made, were not always easy to dissolve.

The impact of contemporary religious sensibilities on Dee’s fortunes and personal outlook can scarcely be overestimated, in a climate hypersensitised by fears of imminent Spanish invasion and Popish plots, uncertainty about the legitimacy of revealed knowledge, and unrest sparked by millennarian anxieties. Like many others, Dee wrestled with the implications for natural philosophy of the impending apocalypse, in which, his angelic guides assured him, he would play a role. While his celebrity and intellectual attainments may have assisted Dee in securing royal audiences, he was not alone in seeking to communicate apocalyptic revelations to European monarchs. Stephen Clucas examines the parallel case of Dee’s correspondent, Roger Edwardes, whose attempts to interest senior divines and officials in his programme for the restitution of the Jews illustrate how popular spiritual anxieties might translate into theological speculation and political correspondence. Edwardes eventually departed England for mainland Europe in search of a more receptive audience: one among the throng of thinkers and writers continually percolating across early modern borders, driven by political or confessional expediency, the hope of employment, the quest for knowledge and the lash of conscience.

From 1583–1589, Dee and his household joined this throng, in obedience to the exhortations of his angelic guides. Dee’s self-imposed exile in East Central Europe was undoubtedly hastened by the poor pickings of Elizabethan patronage and the promise of richer pastures in Poland. In practice, however, it was Dee’s associate, the scryer Edward Kelley (1555–1597), who attracted the attention and investment of continental patrons, including a coveted position at the imperial court of Rudolf II in Prague.

My own essay reassembles archival evidence for Dee and Kelley’s activities in Bohemia, arguing for Dee’s ownership of a long since vanished alchemical manuscript. This document, a copy of the *Bosome book* attributed to the English alchemist George Ripley, was used as a guide to practice by Kelley, who employed this and other works of Ripley to cement his own reputation as an alchemist among his patrons and clients. Kelley’s alchemical prowess soon outstripped that of Dee, providing the basis for his success in building networks of his own. This pattern was repeated back in England, where the abstract formulations of Dee’s alchemical work, the *Monas hieroglyphica* (1564), proved of less interest to Elizabeth and Burghley than the more immediate prospects of transmutation offered by Martin Frobisher, John Prestall and others.^14^

The success of Dee’s *Monas* as a bid for patronage was, at best, indifferent: probably unread by its dedicatee, the Holy Roman Emperor Maximilian II, it had to be explained even to the learned Elizabeth I, and proved too difficult for Maximilian’s successor, Rudolf II. Its opacity was less of a deterrent to scholars, several of whom borrowed from its alchemical and kabbalistic precepts without crediting its author, to Dee’s annoyance (Clulee, 1998; Forshaw, 2005). Andrew Campbell’s case study examines one such unacknowledged connection, in a previously unknown work by the Carmelite friar Paolo Antonio Foscarini (*c.* 1562–1616). The *Monas* provides the basis for eleven theses in Foscarini’s *Scientiarum et artium omnium ferme anacephalaeosis theoretica* (1592), making this work one of the earliest examples of its reception in Italy. In Foscarini’s Christianized interpretation, the *Monas* provides a symbol of the ‘word of God’, while its alchemical importance is downplayed—possibly in response to the outlawing of alchemy by his order. However, in adducing its kabbalistic significance, Foscarini went beyond even Dee, drawing on a range of sources, including Dee’s own authority, the abbot Trithemius of Sponheim.

Alchemy was also a recurring theme in what remains the most problematic dimension of Dee’s networking career: his traffic with angels. The manuscripts that Dee referred to as the *Libri mysteriorum* record his correspondence with non-human and non-material entities, mediated through scryers in his employ, notably Kelley. These ‘actions’ took place within the private spaces of Dee’s household, facilitated by material objects (including the wax tablets and ‘showstones’ discussed by Silke Ackermann and Louise Devoy), and shared with a small circle of privileged acquaintances. The nature of the correspondence at work here, and the extent to which mediums like Kelley subscribed to the authenticity of these transactions, are more difficult to gauge. Dee himself clearly regarded his unseen interlocutors as a source of knowledge for his alchemy, eschatology, and personal and spiritual conduct. His own activities were guided by their instructions, often to a dramatic degree: in the relocation of his household to East Central Europe; his upbraiding of Emperor Rudolf II during his one and only audience; and his acquiescence, however reluctant, to the infamous ‘cross-matching’ between himself, Kelley and their wives. In this regard, Dee sought to establish lines of communication that neither map nor historian can trace—correspondence with Divinity itself.

It is this aspect of Dee’s activity that has captivated the popular imagination from his own day until the present.^15^ Dee was himself the object of collecting practices after his death, as Vittoria Feola shows with reference to the antiquarian Elias Ashmole, who painstakingly garnered Dee’s diaries, records of angel conversations, and other assorted manuscript materials, including horoscopes and designs for magical sigils. However, Feola also draws attention to Ashmole’s collections of Dee’s weather reports: material since overshadowed by the revelations published by Ashmole’s contemporary, Meric Casaubon, as *A true and faithful relation of what passed for many years between Dr John Dee and some spirits* (1659). The material objects that have come to be associated with Dee are also those connected with magic and divination, including a polished obsidian mirror and crystal ball. Ackermann and Devoy survey these and other objects connected with Dee in the British Museum: tracing their provenance back, in some cases, to Dee’s near contemporaries, if not to the man himself.

While these textual and material sources have served to train the modern gaze upon the elusive relations between Dee, Kelley and their angelic guides, they also reflect the interests and activities of other, unseen networks that span the intervening centuries. Collectors and bibliophiles scrambled for Dee’s books and objects even before his death, as evinced by the depredations on Dee’s library during his absence abroad (Roberts & Watson, 1990). Dee’s enduring reputation as magus and conjuror, which he grappled with unsuccessfully during his lifetime, was cemented by the decisions of collectors such as Sir Robert Cotton (1571–1631). Cotton’s interest in Dee’s books and manuscripts, although primarily antiquarian, made possible Casaubon’s edition of the *True and faithful relation* at his grandson John Cotton’s request; the manufacture of the gold disc now held in the British Museum; and the survival of the *Libri mysteriorum*, now held by the British Library.^16^

Modern presentations of Dee might have looked very different had the *Tyrocinium mathematicum* been spared rather than the *Libri*, or had anything survived of Dee’s great collection of scientific instruments, which included instruments and globes made by Frisius and Mercator, and an astrolabe by William Borough.^17^ Yet our present understanding of the breadth of early modern natural philosophical discourse might have been slower in coming without the unavoidable, and sometimes embarrassing, coincidence of Dee’s varied interests, and their galvanizing effect on intellectual historians. Early modern mathematicians and natural philosophers attempted to divine the course of past and future events through perusal of celestial motions, to manipulate matter using alchemical techniques, and to access sacred and universal truths through direct experience of God as well as of nature. The recognition of this intertwining of ‘natural’ and ‘occult’ branches of knowledge has proved both vexing and revelatory for modern cartographers of the early modern sciences. It is at the heart of this tangled correspondence that John Dee comes into his own, as the exemplary practitioner, guide and channel of his age.

## Footnotes

1 Dee (1975), Groundplat. On the *Praeface* see, *inter alia*, Debus, Introduction to Dee (1975), Clulee (1988), pp. 145–176.

2 Dee’s place within the twentieth-century historiography of science receives detailed treatment in Clucas (2006a, 2006b). See also Sherman (1995), pp. 12–26.

3 Various spheres of Dee’s interests of course continue to receive specialist attention. Monographs by Hakan Håkansson (2001) and György E. Szőnyi (2005) study Dee in the context of Renaissance magic and occult philosophy. A recent issue of the *BSHM Bulletin: Journal of the British Society for the History of Mathematics*, *26* (2011) focuses on Dee’s mathematics.

4 Birkbeck College (University of London), 20–21 April 1995 (organised by Stephen Clucas; papers published in Clucas, 2006a); Jozsef Attila University, Szeged, 16–18 July 1998 (organised by György E. Szőnyi); University of Aarhus, 6–9 December 2001 (organised by Jan Bäcklund).

5 *John Dee and alchemy*, Birkbeck College (University of London), 28 May 2005 (organised by Stephen Clucas and the Society for the History of Alchemy and Chemistry); papers published in Clucas (2005).

6 *The John Dee Quatercentenary Conference*, 21–22 September 2009 (organised by Jennifer Rampling, Katie Taylor and Peter Forshaw). On the relatively recent re-dating of Dee’s death to 1609, see Roberts (2004), Roberts & Watson (1990).

7 He was deprived of the latter post under Edward VI. On Rowland Dee, see Parry (2011), chap. 1. I am grateful to Glyn Parry for allowing me to read a draft copy of his forthcoming book, which examines previously neglected archival material relating to Dee and his various networks.

8 Clulee (1988), Harkness (1997), Parry (2011).

9 Dee (1570a, 1570b), Sherman (1995), p. 10.

10 Sherman (1995), p. 7, and Stephen Pumfrey’s essay in this volume. On Elizabethan patronage of natural philosophical expertise more generally, see, inter alia, Pumfrey & Dawbarn (2004), Ash (2004), Harkness (2007).

11 Dee (1851), p. 5. After taking his MA in 1548, Dee returned to Louvain. See Vanden Broecke (2003) , pp. 168–78, 201–12; Parry (2011), chap. 2.

12 On ‘utilitarian’ patronage in England, see Pumfrey & Dawbarn (2004).

13 Dee (1975), sig. A.iij.v. See Clulee (1988, pp. 166–70).

14 The frequently entangled careers of English alchemical practitioners, including the neglected figure of John Prestall, are discussed in Parry (2011); cf. Campbell (2009). On the *Monas*, see Clulee (1988, 2005), Håkansson (2001), Forshaw (2005).

15 These themes were recently restaged in *Dr Dee: An English Opera*, composed by Damon Albarn and directed by Rufus Norris (Palace Theatre, Manchester, 2011).

16 Casaubon (1659) is based on a transcription of British Library MSS Cotton Appendix XLVI, parts I and II.

17 On Borough’s astrolabe, see Baldwin (2006). Another of Dee’s astrolabes was traced by Julian Roberts: Roberts & Watson (1990), Introduction.
